# GABAergic Mechanisms in Schizophrenia: Linking Postmortem and *In Vivo* Studies

**DOI:** 10.3389/fpsyt.2017.00118

**Published:** 2017-08-11

**Authors:** Jeroen C. de Jonge, Christiaan H. Vinkers, Hilleke E. Hulshoff Pol, Anouk Marsman

**Affiliations:** ^1^Brain Center Rudolf Magnus, Department of Psychiatry, University Medical Center Utrecht, Utrecht, Netherlands; ^2^Danish Research Centre for Magnetic Resonance, Copenhagen University Hospital Hvidovre, Hvidovre, Denmark

**Keywords:** GABA, schizophrenia, magnetic resonance spectroscopy, postmortem studies, *in vivo* studies

## Abstract

Schizophrenia is a psychiatric disorder characterized by hallucinations, delusions, disorganized thinking, and impairments in cognitive functioning. Evidence from postmortem studies suggests that alterations in cortical γ-aminobutyric acid (GABAergic) neurons contribute to the clinical features of schizophrenia. *In vivo* measurement of brain GABA levels using magnetic resonance spectroscopy (MRS) offers the possibility to provide more insight into the relationship between problems in GABAergic neurotransmission and clinical symptoms of schizophrenia patients. This study reviews and links alterations in the GABA system in postmortem studies, animal models, and human studies in schizophrenia. Converging evidence implicates alterations in both presynaptic and postsynaptic components of GABAergic neurotransmission in schizophrenia, and GABA may thus play an important role in the pathophysiology of schizophrenia. MRS studies can provide direct insight into the GABAergic mechanisms underlying the development of schizophrenia as well as changes during its course.

## Background

Schizophrenia is a severe chronic psychiatric disorder characterized by hallucinations, delusions, disorganized thinking, and impairments in cognitive functioning, affecting approximately 1% of the population. Several lines of evidence suggest that abnormalities of specific cortical inhibitory neurons and its neurotransmitter γ-aminobutyric acid (GABA) could play an important role in the pathophysiology of schizophrenia (1). The current evidence on GABAergic abnormalities in schizophrenia is mostly based on postmortem studies and has not yet provided a conclusive answer about GABAergic alterations and activity in schizophrenia. *In vivo* measurements of GABA in schizophrenia may reveal additional insights. The aim of this study is to review the findings of postmortem and animal studies on different components of GABAergic neurotransmission and *in vivo* magnetic resonance spectroscopy (MRS) findings on GABA levels in the brains of patients with schizophrenia. To collect relevant literature, a PubMed search was performed using the following terms: ((schizophrenia [tiab] OR schizophrenic* [tiab]) AND (glutamate decarboxylase [tiab] OR glutamic acid decarboxylase [tiab] OR GAD [tiab] OR GAD67 [tiab] OR GAD65 [tiab] OR GABA [tiab] OR gamma-aminobutyric acid [tiab] OR glutamate [tiab] OR glutamergic [tiab] OR gene expression [tiab])).

## Neurobiology of GABA

### Presynaptic GABA Synthesis and Release

**Figure 1:**
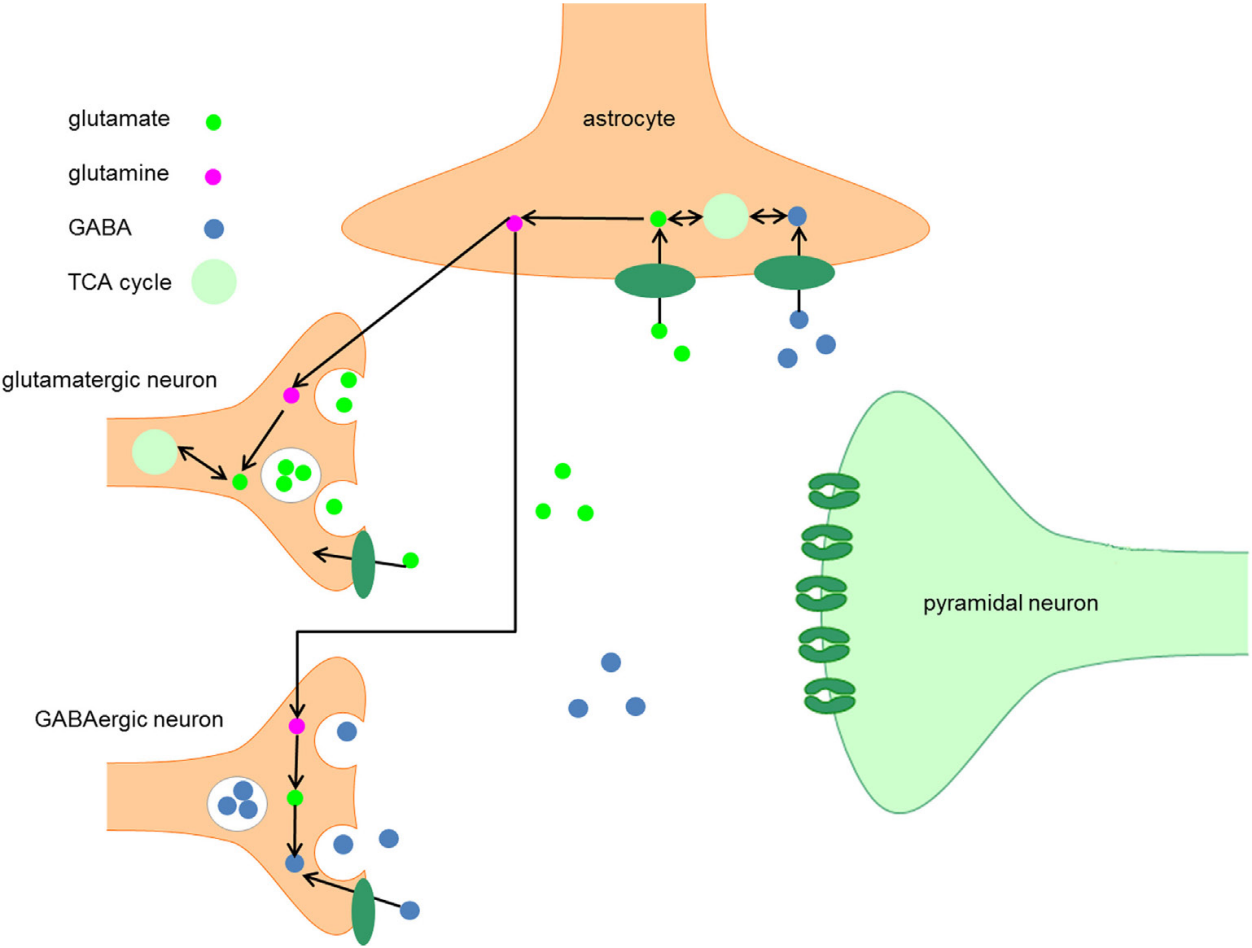
Metabolism of GABA. After synthesis in the presynaptic terminal of GABA neurons, GABA is packaged into vesicles by the vesicular GABA transporter, which is embedded in the vesicular membrane. The synaptic activity of GABA is terminated when GABA is taken up by GABA transporters embedded in the plasma membranes of neurons and astrocytes. When GABA is taken up by neurons, it can be either repacked in vesicles for neurotransmission or it can be degraded by the enzyme GABA transaminase to succinic semialdehyde (SSA). After conversion of SSA to succinate, it enters the TCA cycle and is subsequently converted into glutamate. The following conversion of glutamate to GABA by GAD65 and GAD67 completes the GABA cycle.

GABA is synthesized by decarboxylation of glutamate by glutamic acid decarboxylase (GAD) (Figure 1) (2). Based on its molecular weight, it is possible to distinguish two isotypes, the 65 kDa isotype GAD65 and the 67 kDa isotype GAD67, which are involved in different aspects of GABAergic neurotransmission (3). GAD65 is responsible for rapid synthesis of GABA during periods of high synaptic demand; it is predominantly located on axon terminals and synaptic vesicle membranes and is thus primarily associated with packaging and release of GABA (4–7). GAD67 is responsible for basal GABA levels (4, 5) and the majority (80–90%) of GABA synthesis (8); it is located in the cytosol and is thus primarily associated with GABA synthesis and non-vesicular release (6, 7).

After synthesis in the presynaptic terminal, GABA is packaged into vesicles by the vesicular GABA transporter (VGAT), which is embedded in the vesicular membrane (9). A presynaptic action potential can induce a Ca^2+^-mediated fusion of the vesicle membrane and the presynaptic neuron membrane, which leads to release of GABA into the synaptic cleft. Alternatively, after strong depolarization or altered ion homeostasis, specific GABA transporters (GAT) may reverse their direction resulting in non-vesicular release of GABA (9, 10).

### Postsynaptic GABA Receptors

After release into the synaptic cleft, GABA exerts its inhibitory activity by binding to two types of receptors, such as GABA_A_ and GABA_B_ receptors. GABA_A_ receptors are ligand-gated Cl^−^ channels and produce most of the physiological actions of GABA (11). GABA_A_ receptors have a pentameric subunit structure derived from different gene families and include α, β, γ, δ, ε, π, and θ subunits. Some of these subunits have several isoforms (α1–6, β1–3, and γ1–3) (12). In most cases, the pentamers of subunits include a pair of α subunits and a pair of β subunits in combination with a fifth subunit (γ or δ) (13).

### GABA Transport

The synaptic activity of GABA is terminated when GABA is taken up by GAT that are embedded in the membranes of neurons and astrocytes (10). In humans, four types of GAT can be distinguished, GAT-1 to 3 and the betaine GABA transporter (BGT-1). GAT-1 is widely expressed in the brain, predominantly in presynaptic GABA neurons, and is thus primarily responsible for GABA reuptake (9, 10). GAT-3 is primarily responsible for GABA uptake into local astrocytes (14). In contrast to GAT-1 and GAT-3, GAT-2 and BGT-1 play a very limited role in GABAergic neurotransmission (10).

When GABA is taken up by neurons, it can either be repacked into vesicles or it can be degraded to succinic semialdehyde (SSA) by the enzyme GABA transaminase. After conversion of SSA to succinate, the latter enters the TCA cycle and is subsequently converted into glutamate (10, 15). The following conversion of glutamate to GABA by GAD65 or GAD67 completes the GABA cycle (Figure 1).

## Altered GABAergic Neurotransmission in Schizophrenia

### GAD67 in Schizophrenia

One of the most consistent postmortem findings in schizophrenia is a reduction of mRNA encoding for GAD67 in the dorsolateral prefrontal cortex (DLPFC) in layers 1 through 5 (3–5, 16–29), which results in a reduction of GAD67 protein levels although this has been less extensively studied (Table 1) (4, 30, 31). Since the majority of studies reported unaltered or increased neuronal density, it is unlikely that the reduction of GAD67 mRNA can be attributed to a decrease in the number of neurons in schizophrenia (16, 27, 32, 33). Rather, the density of neurons expressing a detectable level of GAD67 mRNA is decreased (27); expression of GAD67 mRNA is decreased below a detectable level in 25–35% of GABAergic neurons, while the remaining neurons have GAD67 mRNA levels similar to controls (27, 29). It has therefore been suggested that impaired GAD67 gene expression is limited to a certain subset of GABAergic neurons (27, 31). This subset could concern the chandelier, double bouquet, or wide-arbor neurons, which can be distinguished by the presence of specific calcium-binding proteins (Box 1) (1).

**Table 1:** Postmortem studies on glutamic acid decarboxylase (GAD) in schizophrenia.

Reference	Brain region	Findings	Comments
Akbarian et al. (16)	Dorsolateral prefrontal cortex (DLPFC) (BA9)	GAD67 mRNA ↓	
Impagnatiello et al. (34)	Superior temporal gyrus (STG) (BA22)	GAD67 protein ↓	
Benes et al. (35)	Anterior cingulate cortex (ACC) (BA24)	GAD65-IR terminals =	
	DLPFC (BA9)		
Guidotti et al. (30)	DLPFC (BA9)	GAD67 mRNA ↓	Schizophrenia and bipolar disorder
		GAD67 protein ↓	
Mirnics et al. (24)	DLPFC (BA9)	GAD67 mRNA ↓	
Volk et al. (27)	DLPFC (BA9)	GAD67 mRNA ↓	
Hakak et al. (36)	DLFPC (BA46)	GAD 67 mRNA ↑	Elderly patients
		GAD65 mRNA ↑	
Knable et al. (23)	DLPFC (BA9)	GAD67 mRNA ↓	
Hashimoto et al. (5)	DLPFC (BA9)	GAD67 mRNA ↓	
Dracheva et al. (37)	DLPFC (BA46)	GAD67 mRNA ↑	Elderly patients
	Primary visual cortex (VC) (BA17)	GAD65 mRNA ↑	
Woo et al. (28)	ACC (BA24)	GAD67 mRNA ↓	Schizophrenia and bipolar disorder
Hashimoto et al. (19)	DLFPC (BA9)	GAD67 mRNA ↓	
Fatemi et al. (38)	Cerebellar cortex	GAD67 protein ↓	Schizophrenia, bipolar disorder, and major depression
		GAD65 protein ↓	
Veldic et al. (25)	DLPFC (BA9)	GAD67 mRNA ↓	Schizophrenia and bipolar disorder
Straub et al. (39)	DLPFC	GAD67 mRNA ↓	
		GAD67 protein =	
Veldic et al. (26)	DLPFC (BA9)	GAD67 mRNA ↓	
Woo et al. (29)	DLPFC (BA9)	GAD67 mRNA ↓	
Hashimoto et al. (20)	DLFPC (BA9)	GAD67 mRNA ↓	
Hashimoto et al. (21)	DLPFC (BA9)	GAD67 mRNA ↓	
	ACC (BA24)		
	Primary motor cortex		
	Primary VC		
Thompson et al. (3)	ACC (BA24)	GAD67 mRNA ↓ (OFC, caudate, nucleus accumbens)	Schizophrenia, bipolar disorder, and major depression
	Orbital frontal cortex (OFC) (BA45)		
	STG (BA22)		
	Caudate		
	Putamen		
	Nucleus accumbens		
	Medial dorsal thalamus		
	Anterior thalamus		
Duncan et al. (17)	DLPFC (BA9/46)	GAD67 mRNA ↓	
Curley et al. (4)	DLPFC	GAD67 mRNA ↓	
		GAD67 protein ↓	
Kimoto et al. (22)	DLPFC (BA9)	GAD67 mRNA ↓	
Glausier et al. (40)	DLPFC (BA9)	GAD65 mRNA =	
Rocco et al. (31)	DLPFC (BA9)	GAD67 protein ↓	GAD67 protein unaltered in chandelier neurons

Box 1 **Subsets of GABAergic neurons.**

Based on molecular, morphological, and physiological features, it is possible to distinguish different subsets of cortical GABA neurons, with the double bouquet, basket, and chandelier cells being the most abundant cortical GABAergic interneuron subsets (1, 18). The subpopulations have different influences on the regulation of information processing in the dorsolateral prefrontal cortex (DLPFC), partly because the axons of the GABAergic interneurons synapse at different locations on the pyramidal neuron (1, 41, 42). Furthermore, it is possible to identify certain morphological and functional subgroups of GABA neurons which contain different calcium-binding proteins (43–45).

*Chandelier neurons* synapse at axon initial segments (AIS) of pyramidal neurons and therefore provide inhibitory inputs to the AIS. These synaptic connections are formed in such a way that vertical arrays, so-called “cartridges,” are formed (1, 46). Furthermore, these neurons contain the calcium-binding protein *parvalbumin* (5, 47).

*Basket or wide-arbor neurons* synapse at cell bodies and proximal dendrites of pyramidal neurons. Similar to chandelier neurons, basket cells in the prefrontal cortex contain the protein *parvalbumin* (43).

*Double bouquet neurons* contain the calcium-binding protein *calbindin* and target the distal dendrites of pyramidal neurons (1, 48).

A third calcium-binding protein, *calretinin*, is expressed by approximately 50% GABAergic neurons, mainly *double bouquet cells*, in the DLPFC (43).

Since the parvalbumin-containing chandelier and basket neurons synapse at the AIS and soma, respectively, they provide a much stronger inhibitory regulation of the pyramidal neurons as compared to double bouquet cells, which synapse at the distal dendrites (49, 50). Given the heterogeneity in synaptic targets and specific features of the different subclasses of GABAergic neurons, altered interactions between different GABAergic neurons and pyramidal neurons may influence neuronal activity and hence functional output in different manners.

The subset that is affected in schizophrenia appears to include parvalbumin-containing GABAergic neurons. In schizophrenia, parvalbumin mRNA expression is reduced in prefrontal cortex (PFC) layers 3 and 4, but not layers 2, 5, or 6 (5, 27, 29, 51). The overall expression of parvalbumin mRNA is decreased whereas the density of neurons expressing detectable levels of parvalbumin is unaltered (5, 52, 53), implying that the reduction of parvalbumin mRNA is not accompanied by a loss of parvalbumin-containing neurons. The reduced parvalbumin mRNA expression is associated with the decreased density of GAD67 mRNA-positive GABAergic neurons. 50% of the parvalbumin-positive neurons lack detectable amounts of GAD67 mRNA (5), whereas calretinin mRNA (which is expressed by a different subset of neurons—see Box 1) expression and the density of calretinin-positive neurons remain unchanged in schizophrenia (5, 54). These findings imply that the reduced GAD67 mRNA expression may be selective for the parvalbumin-containing subgroup of GABA neurons in the PFC (5). Recent evidence suggests that GAD67 protein levels are unaltered in the chandelier neurons, suggesting that other parvalbumin-containing neurons, such as the basket cells, are involved (31).

The observed alterations regarding parvalbumin are not likely to be caused by exposure to antipsychotic medication. Long-term exposure to haloperidol and benzotropine did not lead to an altered expression of parvalbumin mRNA (5). Furthermore, transcript levels for parvalbumin were reduced to the same extent in the DLPFC of medication-naïve patients compared to patients receiving antipsychotic medication (20). Animal studies have shown that treatment with dopamine D2-receptor antagonists influences the expression of GAD67 mRNA in the basal ganglia (BG) (55–58) but not in the PFC; however, D2-receptor density in the PFC is much lower than in the BG (20, 27).

### GAT-1 in Schizophrenia

The transporter protein GAT-1 is present in the presynaptic neuron and is responsible for the synaptic reuptake of GABA (19, 59). It plays a role in both tonic and phasic GABA-mediated inhibition (60, 61). GAT-1 terminates the synaptic activity of GABA and regulates the duration and efficacy of synaptic GABAergic neurotransmission (62); therefore, reduced GAT-1 levels suggest increased availability of GABA in the synapse (63). Several studies found reduced mRNA levels encoding for the GAT-1 protein in schizophrenia. GAT-1 mRNA levels are decreased in GABAergic neurons in the DLPFC (Table 2) (20, 21, 59, 62, 64). Together with the diminished expression of GAD67 mRNA, it is unclear whether this results in a net increase or decrease of the inhibitory tone on pyramidal cells (63). Moreover, GAT-1 mRNA expression is reduced below detectable levels in a subset of GABAergic neurons and relatively unaltered in the majority of the GABAergic neurons (59). The affected subset appears to include parvalbumin-containing neurons (1, 59). The reduction of GAT-1 mRNA expression is limited to layers 2 through 5, the same layers in which parvalbumin-containing neurons are found (59, 65).

**Table 2:** Postmortem studies on GABA transporters (GAT)-1 in schizophrenia.

Reference	Brain region	Findings
Woo et al. (65)	Dorsolateral prefrontal cortex (DLPFC) (BA9)	GAT-1-IR cartridges of chandelier neurons ↓
Pierri et al. (66)	DLPFC (BA46)	GAT-1-IR cartridges of chandelier neurons ↓
Ohnuma et al. (64)	DLPFC (BA9/10)	GAT-1 mRNA ↓
Volk et al. (59)	DLPFC (BA9)	GAT-1 mRNA ↓
Konopaske et al. (62)	Auditory association area (BA42)	GAT-1-IR cartridges of chandelier neurons =
Hashimoto et al. (20)	DLPFC (BA9)	GAT-1 mRNA ↓
Hashimoto et al. (21)	DLPFC (BA9)	GAT-1 mRNA ↓
	Anterior cingulate cortex (BA24)	
	Primary visual cortex	
	Primary motor cortex	

The subset of GABAergic neurons where reduced GAT-1 mRNA levels are detected is possibly the subset of chandelier neurons (see Box 1). A marker of chandelier neurons is their GAT-1 immunoreactivity; the density of GAT-1 immunoreactive cartridges is decreased in schizophrenia, while markers of other axon terminal populations remain unchanged (65, 66). The lower density of GAT-1 immunoreactive cartridges implies decreased GAT-1 protein, which is associated with decreased GAT-1 mRNA levels. Putting together these findings, reduced GAT-1 mRNA levels may therefore account for the decreased density of GAT-1 immunoreactive axon cartridges in chandelier neurons (59). The reduction of GAT-1 immunoreactive cartridges cannot be attributed to a reduction of chandelier neurons, since the density of GABAergic neurons [identified by parvalbumin (52, 53) and VGAT (67)] is unchanged. Thus concluding, the density of chandelier neurons containing GAT-1 protein in the DLPFC in patients with schizophrenia was reduced whereas the density of parvalbumin-containing neurons remains unaltered. This finding suggests that the reduced levels of GAT-1 mRNA are limited to the chandelier neurons (29, 65).

Long-term exposure to therapeutic blood levels of haloperidol in monkeys did not result in changes in the expression of GAT-1 mRNA or the expression of GAT-1 protein (65, 66, 68), nor did effects of alcohol abuse or benzodiazepine use explain the findings (20, 21).

### Postsynaptic GABA Receptors in Schizophrenia

GABA_A_ receptors are ligand-gated chloride ion channels and produce most of the physiological actions of GABA (11). GABA_A_ receptors have a pentameric subunit structure and the subunits are derived from different gene families encoding for different subunits including α1–6, β1–3, γ1–3, δ, ε, π, and θ (12). The pentamers of subunits include in most cases a pair of α subunits and a pair of β subunits in combination with a fifth subunit (γ or δ) (13). Early studies demonstrated increased binding of muscimol, a selective GABA_A_ receptor agonist, in pyramidal neuronal cell bodies in patients with schizophrenia (69–71); however, muscimol can bind to all types of GABA_A_ receptor subunits. Recent advancements in technology have enabled investigation of deficits of individual GABA_A_ receptor subunits (72).

Subunits of the α-type can be characterized by their subcellular localization within the central nervous system. Over 95% of the GABAergic synapses on the axon initial segment (AIS) of pyramidal neurons contain the α2 subunit, while only 15% of cortical GABA receptors contain the α2 subunit (73, 74). It appears that this subunit is characterized by high affinity, fast activation, and slow deactivation (75). Given its anatomical position and functional features, the GABA_A_ α2 subunit serves as a major source for inhibitory tone on pyramidal neurons (46). Parvalbumin-containing neurons, which appear to exhibit a reduced expression of GAT-1 and GAD67 mRNA in schizophrenia, target the AIS of pyramidal neurons. Indeed, it has been demonstrated that in schizophrenia, the GABA_A_ α2-receptor subunit is upregulated in the AIS of pyramidal neurons (46, 69, 76). This increase in α2 subunit density may occur in response to reduced extracellular GABA concentrations due to diminished GABA synthesis (1, 46). Furthermore, GAT-1 immunoreactive cartridges and the density of α2 subunits at the postsynapse of pyramidal neurons demonstrate an inverse correlation, which implies that GABA_A_ α2 subunits are upregulated at the AIS of pyramidal neurons and GAT-1 is downregulated to provide a synergetic compensation for the diminished GABAergic activity (46). In contrast to GAD67 mRNA and GAT-1 mRNA, mRNA expression levels of postsynaptic GABA_A_ α2-receptor subunits seem to be unaltered (16, 17). Reductions of α2-receptor subunits are exclusively found at the AIS synapses; the lack of upregulation of α2 subunit mRNA might be explained by the fact that inhibitory synapses at the AIS of pyramidal neurons make up less than 10% of the total number of inhibitory synapses of the pyramidal neuron (16, 77).

mRNA levels of the GABA_A_ α1, γ2, α4, α5, and δ receptor subunits are suggested to be downregulated in the DLPFC of patients with schizophrenia (Table 3) (20, 21, 47, 78–80). However, two studies reported an increase of α1 subunit mRNA expression (34, 64), one study revealed an increase of α5 subunit mRNA (34), one study observed an increase of the GABA_A_ receptor α1 subunit protein (72), and one study demonstrated no change of the α4 receptor subunit (80). In contrast to the a2 subunit localized at the AIS of pyramidal neurons, GABA_A_ receptors containing the α1, α5, γ2, and δ (often co-expressed by α4) subunits are predominantly localized in the dendrites of pyramidal neurons (73, 81, 82). The observed alterations in the postsynaptic GABA_A_ receptors do not seem to be a consequence of an increased number of neurons, because the majority of studies have reported no change or an increase in neuron density (27, 32, 33, 51).

**Table 3:** Postmortem studies on postsynaptic GABA receptors in schizophrenia.

Reference	Brain region	Findings
Hanada et al. (71)	Dorsolateral prefrontal cortex (DLPFC) (BA9)	GABA_A_ receptor binding ↑
	Caudate	
Benes et al. (76)	Anterior cingulate cortex (ACC)	GABA_A_ receptor binding ↑
Akbarian et al. (16)	DLPFC (BA9)	GABA_A_ α1–5 receptor subunit mRNA =
		GABA_A_ γ2-receptor subunit mRNA =
Benes et al. (69)	DLPFC (BA10)	GABA_A_ receptor binding ↑
Huntsman et al. (79)	DLPFC (BA9)	GABA_A_ receptor γ2 subunit mRNA ↓
Impagnatiello et al. (34)	DLPFC (BA9)	GABA_A_ receptor α1 subunit mRNA ↑
		GABA_A_ receptor α5 subunit mRNA ↑
Dean et al. (70)	DLPFC (BA9)	GABA_A_ receptor binding ↑
Ohnuma et al. (64)	DLPFC (BA9)	GABA_A_ receptor α1 subunit mRNA ↑
	BA10	
Mirnics et al. (24)	DLPFC (BA9)	GABA_A_ receptor β1, γ2/3, π subunit mRNA ↓
Ishikawa et al. (72)	DLPFC (BA9)	GABA_A_ receptor α1, β2/3 subunit ↑
Ishikawa et al. (83)	DLPFC (BA9)	GABA_B_ receptor 1 protein ↓
Vawter et al. (47)	DLPFC (BA9 + BA46)	GABA_A_ receptor δ subunit mRNA ↓
Volk et al. (46)	Prefrontal cortex	GABA_A_ receptor α2 subunit protein ↑
Hashimoto et al. (20)	DLPFC (BA9)	GABA_A_ receptor α1/4, β3, γ2, δ subunit mRNA ↓
Hashimoto et al. (21)	DLPFC (BA9)	GABA_A_ receptor α1, δ subunit mRNA ↓
	ACC (BA24)	
	Primary visual and motor cortices	
Maldonado-Avilés et al. (80)	DLPFC (BA9)	GABA_A_ receptor δ subunit mRNA ↓
		GABA_A_ receptor α4 subunit mRNA =
Duncan et al. (17)	DLPFC (BA9/BA46)	GABA_A_ receptor α5 subunit mRNA ↓
		GABA_A_ receptor α1/2 subunit mRNA =
Beneyto et al. (78)	DLPFC	GABA_A_ receptor α2 subunit mRNA ↑
		GABA_A_ receptor α1/5, β2 subunit mRNA ↓
		GABA_A_ receptor α3, β1, β3 =

**Figure 2:**
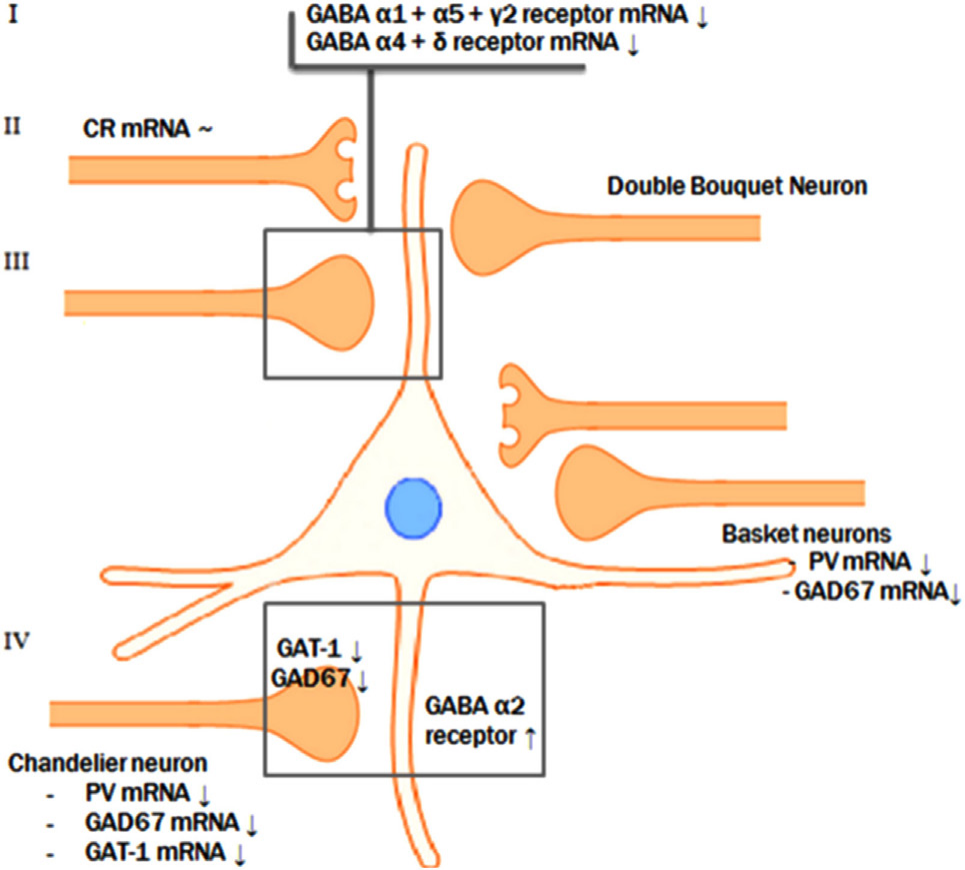
Pre- and postsynaptic GABAergic alterations. The reductions of GAD67 mRNA, parvalbumin mRNA, and GAT-1 mRNA levels in the parvalbumin-containing chandelier neurons seem to result in a compensatory postsynaptic upregulation of α2-receptor at the axon initial segment of the pyramidal neuron. Presynaptic alterations in neurons targeting the dendritic domain of the pyramid neuron might also be accompanied by abnormalities of the postsynaptic GABA α1, α5, and γ2 and the extrasynaptic α4 and δ receptor subunits.

Animal studies in which rats were exposed to benzodiazepines did not reveal changes in the expression level of α2 subunit mRNA or protein levels and long-term exposure to haloperidol or olanzapine did not result in altered α1, α2, α5, β2, or δ subunit mRNA levels (20, 78, 84). Postmortem studies show that α1 and δ subunits are reduced to the same extent in the DLPFC of patients who were not taking antipsychotic medication at the time of death, which is unlikely to be driven by the effects of alcohol abuse or benzodiazepine use (20, 21). (For an overview of pre- and postsynaptic GABAergic alterations, see Figure 2.)

### Widespread GABAergic Alterations in Schizophrenia

There is sufficient histological–pathological evidence to link impairments in GABAergic neurotransmission in other cortical regions than the DLPFC to pathologies and cognitive dysfunctions observed in schizophrenia (63).

Similar to the DLPFC, the anterior cingulate cortex (ACC), primary visual cortex (VC), and primary motor cortex are characterized by the same deficits in GABAergic gene expression as seen in the DLPFC, including selective involvement of parvalbumin-containing subsets of GABA neurons. The largest declines were reported for the levels of mRNA encoding for parvalbumin (21). These brain areas also exhibit a decrease of GAD67 mRNA, GAD65 mRNA, GAT-1 mRNA, and GABA_A_ receptor α1 and δ subunits (1, 21, 28). Calretinin levels remained unchanged (21). GABA-related transcript expression is suggested to be decreased to the same extent in all aforementioned brain regions, so there possibly is no preferential involvement of the DLPFC (21). The reduced expression of GABA_A_ receptor α1 and δ subunits in these cortical areas also imply that reduced phasic and tonic inhibition, respectively, might be a feature shared by multiple cortical regions.

Furthermore, in addition to the ACC, primary VC, and primary motor cortex which demonstrated similar GABAergic expression deficits as the DLPFC, the orbital frontal cortex (OFC), superior temporal gyrus (STG), striatum, and thalamus show a diminished GAD67 mRNA expression as well (3). In addition, the STG and auditory gyri demonstrated reduced GAT-1 protein levels (34). Reduction in GABAergic activity in the OFC could lead to disturbances related to emotional and cognitive functioning and may therefore underlie symptoms regarding social withdrawal and apathetic behavior (85). In addition, abnormalities in the STG could contribute to deficit auditory processing and auditory hallucinations (3). These findings imply that the aberrations seen in the DLPFC may not be due to alterations in DLPFC circuitry only, but that the altered transcript levels appear to be the consequence of a common upstream mechanism that operates across multiple cortical areas.

### Integration of Postmortem Findings on GABAergic Neurotransmission

A possible integrative model for the alterations in GABA neurotransmission is that a subset of prefrontal GABA neurons is affected in schizophrenia. In contrast to the reduced GAD67 and the consequent attenuation of inhibitory GABAergic neurotransmission, the reduction of GAT-1 mRNA expression tends to increase the synaptic activity of GABA (63). In addition, GABA_A_ receptors are upregulated in postsynaptic pyramidal neurons, which suggests a compensatory increase in response to the decreased extracellular GABA concentrations (46, 70, 76). However, based on postmortem studies, it is not possible to identify the initial deficit in the pathological chain and, therefore, two scenarios are possible (see Figure 2).

The most likely scenario is an overall reduced GABAergic activity in schizophrenia. This implies that the initial step in this specific pathologic process is the presynaptic reduction of GABA synthesis, followed by a secondary, compensatory reduction of reuptake by means of GAT-1 and by compensatory upregulation of postsynaptic GABA receptors (1, 18, 86). This synergetic attempt, to improve the GABAergic neurotransmission at the synapse of the pyramidal neuron AIS, serves to compensate for the initial deficit in synthesis of GABA. Consistent with the theory that the reduction of synthesis is the first step in the pathological chain, mice lacking the GAT-1 gene do not develop diminished levels of GAD67 mRNA. This indicates that the reduction of GAD67 is the initial event (87). Furthermore, GABA hypofunction due to decreased synthesis reflected by the diminished levels of GAD67 mRNA was imitated in rats by means of pharmacological blockade of prefrontal GABA_A_ receptors. This resulted in impaired working memory performance, a cognitive function characteristically disturbed in patients with schizophrenia (88, 89). However, it is still controversial whether the compensatory mechanisms are sufficient to overcome the decreased GABA synthesis. In other words, it is unknown if the net effect of the diminished presynaptic synthesis on the one hand and the decreased reuptake increased postsynaptic reception on the other hand result in an increase or decrease of the inhibitory tone on pyramidal cells by GABAergic neurons (63). In conclusion, the most likely scenario is that reduced presynaptic GABA production results in a reduced reuptake of GABA and in upregulated postsynaptic GABA receptors in schizophrenia.

Alternatively, an excessive increase of GABAergic activity due to both primary diminished reuptake and upregulated postsynaptic receptors may also be an initial step in the pathological process followed by secondary compensatory downregulation of GAD67 mRNA in chandelier neurons due to the excessive GABAergic activity. Furthermore, the effects of pharmaceuticals involved in GABAergic neurotransmission seem to be in line with the hypothesis of excess GABAergic activity. For example, lorazepam, a positive allosteric modulator of GABAergic neurotransmission, results in a deterioration of working memory aberrations while flumazenil, a partial inverse agonist, leads to improvement of the working memory deficits (63). Thus, according to this scenario, excessive GABAergic activity could be the result of an initial postsynaptic upregulation of the GABA_A_ receptor and downregulation of the presynaptic GABA reuptake transporters as a first step in the pathological chain (63).

Finally, the aberrations seen in the DLPFC may not be due to alterations in DLPFC circuitry, but instead reflect transcript levels that are a consequence of a common upstream mechanism that operates across multiple cortical areas in schizophrenia.

In conclusion, the most likely scenario involves reduced GABA concentrations due to a compromised production of GABA reflected by the diminished concentration of GAD67 mRNA. However, due to the observation that presynaptic GAT-1 is reduced and postsynaptic receptors are upregulated, postmortem studies do not provide a conclusive answer about the net GABAergic concentrations and activity. Therefore, *in vivo* studies could provide additional insights into GABA levels in clinical states contributing to a more definitive formulation about the pathological cascade and GABAergic alterations in schizophrenia.

## *In Vivo* MRS of GABA in Schizophrenia

GABA can be measured *in vivo* using proton MRS (^1^H-MRS). MRS provides a means to non-invasively identify and quantify metabolites in tissue and can be carried out with an MR scanner. MRS makes use of the magnetic properties of nuclei, e.g., the proton (^1^H). Because the magnetic properties of a nucleus are influenced by its chemical environment, it is possible to identify signals from different molecules within the MR spectrum. However, measurement of GABA with ^1^H-MRS is challenging since its low concentration results in a relatively small signal which is overlapped by more intense signals from more abundant metabolites. It is possible to separate the GABA signal from other, more intense signals with spectral editing techniques. With spectral editing the magnetic properties of a specific molecule are used to improve detection of that molecule.

Based on presynaptic and postsynaptic GABAergic alterations in postmortem studies, it is possible to identify numerous brain areas such as the ACC, primary VC, primary motor cortex, OFC, BG, STG, thalamus, but especially the DLPFC in which it is expected to measure altered GABAergic concentrations by ^1^H-MRS. As mentioned before, postmortem studies do not provide a conclusive answer about the net GABAergic concentrations and activity. Therefore, ^1^H-MRS could provide additional insights, contributing to a more definitive formulation about the pathological cascade and GABAergic alterations in schizophrenia. However, up until now MRS studies on GABA in schizophrenia are rather scarce and only cross-sectional. Moreover, the current literature is inconsistent regarding the measured GABA levels in different brain regions of patients with schizophrenia. Currently, seven studies reported GABA reductions (90–96), six studies reported unchanged GABA levels (90, 92–94, 97, 98), and two studies reported increased levels (Table 4) (97, 99). Since GABA levels may differ in early (90, 91, 93, 94) and chronic schizophrenia (91, 93, 94, 98, 99), brain levels might also be dependent on the stage of the disease. Recent meta-analysis showed no changes in GABA levels in patients with schizophrenia in any given brain region, however, when averaging GABA levels across all measured brain regions per study, GABA appeared to be lower in patients compared to healthy controls (100).

**Table 4:** *In vivo* magnetic resonance spectroscopy studies of GABA in schizophrenia.

Reference	Findings			Antipsychotic medication, % of patients			Comments
	Early SZ	Chronic SZ	Mixed population	Early SZ	Chronic SZ	Mixed population	
Goto et al. (90)	ACC:			Atypical 100% (risperidone, olanzapine, aripiprazole, quetiapine)			Patients were examined at baseline and after 6 months of antipsychotic treatment
	baseline =						
	6M =						
	baseline–6M =						
	BG:						
	baseline ↓						
	6M ↓						
	baseline–6M =						
	POC:						
	baseline =						
	6M =						
	baseline–6M =						

Ongur et al. (99)		ACC ↑			Unknown 100%		1 early SZ patient (0.5%)
		POC ↑					

Tayoshi et al. (98)		ACC =			Typical ± atypical 42%		
		BG =			Atypical only 58%		

Yoon et al. (96)			VC ↓			Typical 8%	
						Atypical 54%	
						Unmedicated 38%	

Kegeles et al. (97)			MPFC:	Atypical 100%	Typical 20%		
			unmed. ↑				
			med. =				
			unmed.–med. =				
			DLPFC:		Atypical 80%		
			unmed. =				
			med. =				
			unmed.–med. =				

Kelemen et al. (91)	VC:			Typical 11%			Patients were examined at baseline and after 6 months of antipsychotic treatment
	baseline ↓			Atypical 89%			
	6M ↓						

Marsman et al. (92)			PFC ↓			Atypical 100%	Min.–max. disease duration: 1–213 months
			POC =				

Rowland et al. (93)	ACC =	ACC ↓		Atypical 100%	Typical 20%		
		ACC early-chronic =			Atypical 80%		
	CSO =	CSO =					
		CSO early-chronic =					

Rowland et al. (94)	ACC =	ACC ↓		Typical 3.5%	Typical 13%		
				Atypical 86%	Atypical 58%		
		ACC early-chronic ↓		Typical + atypical 3.5%	Typical + atypical 19%		
				Unmedicated 7%	Unmedicated 10%		

Wang et al. (95)	PFC ↓			Drug naïve 100%			All first-episode SZ

*ACC, anterior cingulate cortex; BG, basal ganglia; POC, parieto-occipital lobe; PFC, prefrontal cortex; MPFC, medial prefrontal cortex; DLPFC, dorsolateral prefrontal cortex; CSO, centrum semiovale; VC, visual cortex*.

The fluctuating and inconsistent findings of the few MRS studies that have been published so far in schizophrenia could be explained by several factors such as small and heterogeneous sample sizes, low magnetic field strengths resulting in a less robust measurement of GABA, methodological limitations leading to relatively large voxel volumes and marginal adjustments with regard to gray and white matter differences (Table 4) (15). Moreover, most studies measured GABA referenced to creatine and although this is a common approach, fluctuations in creatine concentrations could be, to a certain extent, responsible for the observed GABAergic findings. However, the most prominent limitation compromises the undetermined role of antipsychotic medication use with regard to GABA levels measured by ^1^H-MRS.

## GABA and Antipsychotic Medication

In 38 chronic schizophrenia patients, higher GABA concentrations were found in the left BG in patients using typical antipsychotics as compared to patients using atypical antipsychotics (82). Furthermore, a positive correlation was reported between GABA concentration in the left BG and anticholinergic medication (98). It is thus possible that antipsychotic medication influences GABA concentrations and different types of medications could have differing effects (98).

However, in patients diagnosed with schizophrenia and using antipsychotic medication at baseline, the use of atypical antipsychotics did not have any effects on GABA concentrations in the left BG, frontal lobe, and parieto-occipital lobe during a follow-up period of 6 months (90). At baseline, the concentration of GABA in the left BG in these first-episode patients was decreased (81), but this reduction was not reversed after 6 months of treatment with antipsychotic medication (84). Interestingly, clinical condition, assessed by PANSS scores, did improve during this time period. This suggests that medication use has no profound effect on GABA concentrations in patients with schizophrenia although there does occur a clinical improvement (90, 101). However, it is also possible that the medication regimen prevented further progressive reduction of GABA concentrations in these patients. Studying patients not taking antipsychotic medication may provide valuable additional insights regarding this matter. A recent study addressed this topic and evaluated GABA concentration in 16 unmedicated patients, consisting of 9 medication-naïve patients and 7 patients with no antipsychotic medication use 14 days prior to the investigation. This study observed higher GABA concentrations in never- and unmedicated patients compared to medicated patients (97). This implies that medication use might lead to a normalization of GABA concentrations (97). However, as mentioned before, medicated patients did not show any alterations regarding GABA concentrations after 6 months of antipsychotic therapy (90). Possibly, patients that were minimally treated at baseline differed from those that were medication naïve (90, 101), and the normalization of GABA concentrations due to antipsychotic treatment takes place at the beginning of the treatment. To formulate a conclusive answer, future studies are required, which assess both within-subject medication and medication-naïve study designs. In conclusion, many factors contribute to the inconsistency in literature and future studies need to take these factors into account to reconcile the fluctuating findings.

## GABA and Cognition

The observed changes in GABAergic neurotransmission may have functional significance (96). GABA measurement in the VC revealed reduced concentrations, and this decrease was positively correlated with orientation-specific surround suppression (OSSS) (96). OSSS is a behavioral measure of visual inhibition, and it is believed that this process relies on GABAergic neurotransmission in the VC (85). Furthermore, poorer performance on attention tests was correlated with decreased GABA concentrations in patients with schizophrenia (93). These observations are consistent with the GABA deficit hypothesis, which states that reduced GABAergic neurotransmission results in cognitive deficits, and imply that MRS is able to measure the pool of cortical GABA that has a direct relationship with GABA-mediated functions (15). Since the GABAergic expression deficits exhibit a widespread cortical involvement, it is likely that such aberrations generalize to other cortical areas (21, 96).

On the other hand, recent research showed a negative association between level of cognitive functioning and GABA level in the PFC in schizophrenia patients (92). Together with the finding that GABA levels are reduced in schizophrenia and albeit the finding that intelligence levels are lower in patients compared to matched healthy controls (102), this may imply that the GABA deficit hypothesis mainly applies to patients with lower intelligence (92). Alternatively, patients with higher intelligence may have better treatment compliance, possibly resulting in lower GABA levels (92).

## Integrating Postmortem and *In Vivo* GABA Findings in Schizophrenia

The reported elevation of GABA levels in the MPFC by ^1^H-MRS in unmedicated patients seems to be inconsistent with the results of postmortem studies, which exhibit an impaired GABA synthesis of parvalbumin-containing subclasses of GABA neurons reflected by diminished GAD67 mRNA levels (97). This discrepancy could be explained by the extensive exposure of the postmortem brain samples to antipsychotic medication in predominantly chronically ill patients (18). Furthermore, the observed elevated GABA levels in the MPFC might also be an overcompensation of other subclasses of GABA neurons (97). The NMDA-receptor hypofunction hypothesis puts forward that an intrinsic deficit of GABA neurons, including impaired GABA synthesis, results in disinhibition of pyramidal neurons. The deficit regulation of pyramidal neurons by GABAergic neurotransmission leads to glutamate elevations (48, 103). Therefore, the remaining unimpaired subclasses (subclasses other than the parvalbumin-containing subclass) could be stimulated by the increased glutamergic activity, and this could serve as a compensation for the diminished synthesis in the parvalbumin-containing subclass (97).

Recent advantages in ultrahigh-field MR techniques allow for a more robust assessment of GABA levels, and future studies must point out whether *in vivo* measurement of GABA corresponds with the observed GABA deficiencies in postmortem tissues and whether the GABAergic deficits occur in a pan-cortical manner. Moreover, futures studies might point out if GABA concentrations predict functional outcome and if alterations in GABA concentrations relate to therapy response. It is clear that GABA measurement by *in vivo* MR spectroscopy could be of great value, but it is also evident that further work is needed to provide additional information on the validation of MR spectroscopy of GABA in schizophrenia.

## Conclusion

Converging evidence implicates alterations in both presynaptic and postsynaptic components of GABA neurotransmission to fulfill an important role in the pathophysiology of schizophrenia. Multiple research sites using *in situ* hybridization, DNA microarray, or real-time quantitative PCR have consistently found reduced levels of GAD67 mRNA or a reduced density of neurons positive for GAD67 mRNA in the DLPFC as one of the most consistent findings with regard to pathological changes in schizophrenia. This decrease is the consequence of a reduction of GAD67 mRNA in a subset of GABA neurons. The affected neurons appear to include the parvalbumin-containing neurons. Parvalbumin-positive cells in the DLPFC include chandelier cells, targeting the upregulated α2-receptor subunit at the AIS of the pyramidal neuron. Furthermore, since GAD67 mRNA expression deficits were also observed in layers without parvalbumin expression, other subclasses may attribute to the observed GABAergic gene expression deficits as well. Furthermore, since other brain regions demonstrated similar GABAergic gene expression deficits as the DLPFC, disturbances in GABAergic neurotransmission could be the consequence of a common upstream effect. Therefore, identifying a common pathophysiology might give rise to new pharmacological opportunities in the treatment of schizophrenia. Measurement of GABA levels *in vivo* by means of MRS offers the possibility to approach the illness from a unique perspective and provides additional insights in the relationship between deficit components of GABA neurotransmission and GABA-mediated inhibitory activity. However, the current literature is inconsistent regarding the measured GABA levels in different brain regions of patients with schizophrenia. Future MRS studies using GABA editing are required to give us a better understanding of the pathophysiology of schizophrenia in different stages of the disease. Particularly GABA-editing at ultrahigh-field strengths will be beneficial for detection of the relatively small GABA signal, because of the increased sensitivity, resolution, and signal-to-noise ratio, allowing for an accurate and time-efficient assessment of GABA levels.

## Author Contributions

JJ contributed to the design of the study, performed literature research, and wrote and prepared the manuscript. CV contributed to the writing of the manuscript. HH contributed to the design of the study and the writing of the manuscript. AM contributed to the design of the study and supervised and contributed to the literature research, writing, and preparation of the manuscript.

## Conflict of Interest Statement

The authors declare that the research was conducted in the absence of any commercial or financial relationships that could be construed as a potential conflict of interest.
